# Supplementary material for: A Critical View of the Application of the APEX Software (Aqueous Photochemistry of Environmentally-Occurring Xenobiotics) to Predict Photoreaction Kinetics in Surface Freshwaters
Source: Molecules. 2019 Dec 18;25(1):9. doi: 10.3390/molecules25010009 (PMC7017383; doi:10.3390/molecules25010009)
Supplement: Supplementary file 1 [file molecules-25-00009-s001.zip › 3.2.4_gcc-4.4.0.zip!/3.2.4_gcc-4.4.0/3.2.4_gcc-4.4.0/doc/octave/html/interpreter/Getting-Help.html]

Getting Help - Untitled


Next: Command Line Editing,
Previous: Quitting Octave,
Up: Getting Started


---

### 2.3 Commands for Getting Help

The entire text of this manual is available from the Octave prompt
via the command `doc`. In addition, the documentation for
individual user-written functions and variables is also available via
the `help` command. This section describes the commands used for
reading the manual and the documentation strings for user-supplied
functions and variables. See Function Files, for more information
about how to document the functions you write.

— Command: help name  
> Display the help text for name.
> If invoked without any arguments, `help` prints a list
> of all the available operators and functions.
>
> For example, the command `help help` prints a short message
> describing the `help` command.
>
> The help command can give you information about operators, but not the
> comma and semicolons that are used as command separators. To get help
> for those, you must type `help comma` or `help semicolon`.
>
>
>
> **See also:** doc, lookfor, which.

— Command: doc function\_name  
> Display documentation for the function function\_name
> directly from an on-line version of
> the printed manual, using the GNU Info browser. If invoked without
> any arguments, the manual is shown from the beginning.
>
> For example, the command `doc rand` starts the GNU Info browser
> at the `rand` node in the on-line version of the manual.
>
> Once the GNU Info browser is running, help for using it is available
> using the command `C-h`.
>
>
>
> **See also:** help.

— Command: lookfor str  
— Command: lookfor **-all** str  
— Function: [func, helpstring] = **lookfor** (str)  
— Function: [func, helpstring] = **lookfor** ('-all', str)  
> Search for the string str in all functions found in the current
> function search path. By default, `lookfor` searches for str
> in the first sentence of the help string of each function found. The entire
> help text of each function can be searched if the '-all' argument is
> supplied. All searches are case insensitive.
>
> Called with no output arguments, `lookfor` prints the list of
> matching functions to the terminal. Otherwise, the output arguments
> func and helpstring define the matching functions and the
> first sentence of each of their help strings.
>
> The ability of `lookfor` to correctly identify the first
> sentence of the help text is dependent on the format of the
> function's help. All Octave core functions are correctly
> formatted, but the same can not be guaranteed for external packages and
> user-supplied functions. Therefore, the use of the '-all' argument may
> be necessary to find related functions that are not a part of Octave.
>
>
>
> **See also:** help, doc, which.

To see what is new in the current release of Octave, use the `news`
function.

— Function File: **news** ()  
> Display the current NEWS file for Octave.

— Function File: **info** ()  
> Display contact information for the GNU Octave community.

— Built-in Function: **warranty** ()  
> Describe the conditions for copying and distributing Octave.

The following functions can be used to change which programs are used
for displaying the documentation, and where the documentation can be
found.

— Built-in Function: val = **info\_file** ()  
— Built-in Function: old\_val = **info\_file** (new\_val)  
> Query or set the internal variable that specifies the name of the
> Octave info file. The default value is
> octave-home/info/octave.info, in
> which octave-home is the root directory of the Octave installation.
> The default value may be overridden by the environment variable
> `OCTAVE_INFO_FILE`, or the command line argument
> --info-file NAME.
>
>
>
> **See also:** info\_program, doc, help, makeinfo\_program.

— Built-in Function: val = **info\_program** ()  
— Built-in Function: old\_val = **info\_program** (new\_val)  
> Query or set the internal variable that specifies the name of the
> info program to run. The default value is
> octave-home/libexec/octave/version/exec/arch/info
> in which octave-home is the root directory of the Octave installation,
> version is the Octave version number, and arch
> is the system type (for example, `i686-pc-linux-gnu`). The
> default value may be overridden by the environment variable
> `OCTAVE_INFO_PROGRAM`, or the command line argument
> --info-program NAME.
>
>
>
> **See also:** info\_file, doc, help, makeinfo\_program.

— Built-in Function: val = **makeinfo\_program** ()  
— Built-in Function: old\_val = **makeinfo\_program** (new\_val)  
> Query or set the internal variable that specifies the name of the
> program that Octave runs to format help text containing
> Texinfo markup commands. The default value is `makeinfo`.
>
>
>
> **See also:** info\_file, info\_program, doc, help.

— Built-in Function: val = **doc\_cache\_file** ()  
— Built-in Function: old\_val = **doc\_cache\_file** (new\_val)  
> Query or set the internal variable that specifies the name of the
> Octave documentation cache file. A cache file significantly improves
> the performance of the `lookfor` command. The default value is
> octave-home/share/octave/version/etc/doc-cache,
> in which octave-home is the root directory of the Octave installation,
> and version is the Octave version number.
> The default value may be overridden by the environment variable
> `OCTAVE_DOC_CACHE_FILE`, or the command line argument
> --doc-cache-file NAME.
>
>
>
> **See also:** lookfor, info\_program, doc, help, makeinfo\_program.

— Built-in Function: val = **suppress\_verbose\_help\_message** ()  
— Built-in Function: old\_val = **suppress\_verbose\_help\_message** (new\_val)  
> Query or set the internal variable that controls whether Octave
> will add additional help information to the end of the output from
> the `help` command and usage messages for built-in commands.
